# Information Theory for Data Communications and Processing

**DOI:** 10.3390/e22111250

**Published:** 2020-11-03

**Authors:** Shlomo Shamai (Shitz), Abdellatif Zaidi

**Affiliations:** 1 The Viterbi Faculty of Electrical Engineering, Technion—Israel Institute of Technology, Haifa 3200003, Israel; 2 Institut Gaspard Monge, Université Paris-Est, 05 Boulevard Descartes, Cité Descartes, 77454 Champs sur Marne, France

**Keywords:** information theory, data communications, data processing

This book, composed of the collection of papers that have appeared in the Special Issue of the *Entropy* journal dedicated to “Information Theory for Data Communications and Processing”, reflects, in its eleven chapters, novel contributions based on the firm basic grounds of information theory. The book chapters [1,2,3,4,5,6,7,8,9,10,11] address timely theoretical and practical aspects that carry both interesting and relevant theoretical contributions, as well as direct implications for modern current and future communications systems.

Information theory has started with the monumental work of Shannon: Shannon, C.E. “A Mathematical Theory of Communications”, *Bell System Technical Journal*, vol. 27, pp. 379–423, 623–656, 1948, and it provided from its very start the mathematical/theoretical framework which facilitated addressing information related problems, in all respects: starting with the basic notion of what is information, going through basic features of how to convey information in the best possible way and how to process it given actual and practical constraints. Shannon himself not only fully realized the power of the basic theory he has developed but further in his profound contributions addressed practical constraints of communications systems, such as bandwidth, possible signaling limits (as peak limited signals), motivating from the very start to address practical constraints via theoretical tools, see, for example: Jelonek, Z. A comparison of transmission systems, In Proc. Symp. Appl. Commun. Theory, E.E. Dep., Imperial College, Buttenvorths Scientific Press, London, September 1952, pp. 45–81. Shannon has contributed fundamentally also to most relevant aspects as source coding under a fidelity (distortion) measure, finite code lengths (error exponents) as well as network aspects of information theory (the multiple-access channel), see: Sloane, N.J.A. and Wyner, A.D., Eds., Collected Papers of Claude Elwood Shannon. IEEE Press: New York, 1993.

While at its beginning and through the first decades, information theory, as is reflected in the basic 1948 work of Shannon, was a mathematical tool that pointed out the best that can be achieved (as channel capacity for point-to-point communications), which with past technology could not even be imagined to be approached. Now, the power of information theory is way greater as it is able to theoretically address network problems and not only point out the limits of communications/signal processing, but with current technology, those limits can, in general, be decently approached. This is classically demonstrated by the capacity of the point-to-point Gaussian channel, which is actually achieved within fractions of dB in signal-to-noise (snr) ratio by advanced coding techniques (Low-Density-Parity-Check, Turbo and Polar codes). In our times, current advanced technology turns information theory into a practical important tool that is capable also to provide basic guidelines how to come close to ultimate optimal performance.

Modern, current and future communications/processing aspects motivate in fact basic information theoretic research for a wide variety of systems for which we yet do not have the ultimate theoretical solutions (for example a variety of problems in network information theory as the broadcast/interference and relay channels, which mostly are yet unsolved in terms of determining capacity regions and the like). Technologies as 5/6G cellular communications, Internet of Things (IoT), Mobile Edge Networks and others place in center not only features of reliable rates of information measured by the relevant capacity, and capacity regions, but also notions such as latency vs. reliability, availability of system state information, priority of information, secrecy demands, energy consumption per mobile equipment, sharing of communications resource (time/frequency/space) and the like.

This book focuses on timely and relevant features, and the contributions in the eleven book chapters [1,2,3,4,5,6,7,8,9,10,11], summarized below, address the information theoretical frameworks that have important practical implications.

The basic contributions of this book could be divided into three basic parts:

(1) The first part Chapters [1,2,3,4,5] considers central notions such as the Information Bottleneck, overviewed in the first chapter, pointing out basic connections to a variety of classical information theoretic problems, such as remote source coding. This subject covering timely novel information theoretic results demonstrates the role information theory plays in current top technology. These chapters, on one hand, provide application to ‘deep learning’, and, on the other, they present the basic theoretical framework of future communications systems such as Cloud and Fog Radio Access Networks (CRAN, FRAN). The contributions in this part directly address aspects such as ultra-reliable low-latency communications, impacts of congestion, and non-orthogonal access strategies.
(2) The second part of the contributions in this book Chapters [6,7,8] addresses classical communications systems, point-to-point Multiple-Input-Multiple-Output (MIMO) channels subjected to practical constraints, as well as network communications models. Specifically, relay and multiple access channels are discussed.
(3) The third part of the contributions of this book Chapters [9,10,11] focuses mainly on caching, which, for example, is the center component in FRAN. Information theory indeed provides the classical tool to address network features of caching, as demonstrated in the contributions summarized below (and references therein).

Chapter 1: “On the Information Bottleneck Problems: Models, Connections, Applications and Information Theoretic Views” provides a tutorial that addresses from an information theoretic viewpoint variants of the information bottleneck problem. It provides an overview emphasizing variational inference, representation learning and presents a broad spectrum of inherent connections to classical information theoretic notions such as: remote source-coding, information combining, common reconstruction, the Wyner–Ahlswede–Korner problem and others. The distributed information bottleneck overviewed in this tutorial sets the theoretical grounds for the uplink CRAN, with oblivious processing.

Chapter 2: “Variational Information Bottleneck for Unsupervised Clustering: Deep Gaussian Mixture Embedding” develops an unsupervised generative clustering framework that combines the variational information bottleneck and the Gaussian mixture model. Among other results, this approach that models the latent space as a mixture of Gaussians generates inference-type algorithms for exact computation, and generalizes the so-called evidence lower bound, which is useful in a variety of unsupervised learning problems.

Chapter 3: “Asymptotic Rate-Distortion Analysis of Symmetric Remote Gaussian Source Coding: Centralized Encoding vs. Distributed Encoding” addresses remote multivariate source coding, which is a CEO problem and, as indicated in Chapter 1, connects directly to the distributed bottleneck problem. The distortion measure considered here is minimum-mean-square-error, which can be connected to the logarithmic distortion via classical information–estimation relations. Both cases—the distributed and joint remote source coding (all terminals cooperate)—are studied.

Chapter 4: “Non-Orthogonal eMBB-URLLC Radio Access for Cloud Radio Access Networks with Analog Fronthauling” provides an information-theoretic perspective of the performance of Ultra-Reliable Low-Latency Communications (URLLC) and enhanced Mobile BroadBand (eMBB) traffic under both Orthogonal and Non-Orthogonal multiple access procedures. The work here considers CRAN based on the relaying of radio signals over analog fronthaul links.

Chapter 5: “Robust Baseband Compression against Congestion in Packet-Based Fronthaul Networks Using Multiple Description Coding” also addresses CRAN and considers the practical scenario when the fronthaul transport network is packet based and it may have a multi-hop architecture. The timely information theoretic concepts of multiple description coding are employed, and demonstrated to provide advantageous performance over conventional packet-based multi-route reception or coding.

Chapter 6: “Amplitude Constrained MIMO Channels: Properties of Optimal Input Distributions and Bounds on the Capacity” studies the classical information theoretic setting where input signals are subjected to practical constraints, with focus on amplitude constraints. Followed by a survey of available results for Gaussian MIMO channels, which are of direct practical importance, it is shown that the support of a capacity-achieving input distribution is a small set in both a topological and a measure theoretical sense. Bounds on the respective capacities are developed and demonstrated to be tight in the high amplitude regime (high snr).

Chapter 7: “Quasi-Concavity for Gaussian Multicast Relay Channels” addresses the classical model of a relay channel, which is one of the classical information theoretic problems that are not yet fully solved. This work identifies useful features of quasi-concavity of relevant bounds (as the cut-set bound) that are useful in addressing communications schemes based on relaying.

Chapter 8: “Gaussian Multiple Access Channels with One-Bit Quantizer at the Receiver” investigates the practical setting when the received input is sampled and here it employs a zero-threshold one-bit analogue-to-digital converter. It is shown that the optimal capacity achieving signal distribution is discrete, and bounds on the respective capacity are reported.

Chapter 9: “Efficient Algorithms for Coded Multicasting in Heterogeneous Caching Networks” addresses crucial performance–complexity tradeoffs in a heterogeneous caching network setting, where edge caches with possibly different storage capacities collect multiple content requests that may follow distinct demand distributions. The basic known performance-efficient coded multicasting schemes suffer from inherent complexity issues, which makes them impractical. This chapter demonstrates that the proposed approach provides a compelling step towards the practical achievability of the promising multiplicative caching gain in future-generation access networks.

Chapter 10: “Cross-Entropy Method for Content Placement and User Association in Cache-Enabled Coordinated Ultra-Dense Networks” focuses on ultra-dense networks, which play a central role for future wireless technologies. In Coordinated Multi-Point-based Ultra-Dense Networks, a great challenge is to tradeoff between the gain of network throughput and the degraded backhaul latency, and caching popular files has been identified as a promising method to reduce the backhaul traffic load. This chapter investigated Cross-Entropy methodology for content placement strategies and user association algorithms for the proactive caching ultra-dense networks, and demonstrates advantageous performance.

Chapter 11: “Symmetry, Outer Bounds, and Code Constructions: A Computer-Aided Investigation on the Fundamental Limits of Caching” also focuses on caching, which, as mentioned, is a fundamental procedure for future efficient networks. Most known analyses and bounds developed are based on information theoretic arguments and techniques. This work illustrates how computer-aided methods can be applied to investigate the fundamental limits of the caching systems, which are significantly different from the conventional analytic approach usually seen in the information theory literature. The methodology discussed and suggested here allows, among other things, to compute performance bounds for multi-user/terminal schemes, which were believed to require unrealistic computation scales.

In closing, one can view all the above three categories of the eleven chapters, in a unified way, as all are relevant to future wireless networks. The massive growth of smart devices and the advent of many new applications dictates not only having better systems, such as coding and modulation on the point-to-point channel, classically characterized by channel capacity, but a change of the network/communications paradigms (as demonstrated for example, by the notions of CRAN and FRAN) and performance measures. New architectures and concepts are a must in current and future communications systems, and information theory provides the basic tools to address these, developing concepts and results, which actually are not only of essential theoretical value, but are also of direct practical importance. We trust that this book provides a sound glimpse to these aspects.
